# Supplementary material for: ARA: accurate, reliable and active histopathological image classification framework with Bayesian deep learning
Source: Sci Rep. 2019 Oct 4;9:14347. doi: 10.1038/s41598-019-50587-1 (PMC6778075; doi:10.1038/s41598-019-50587-1)
Supplement: Supplementary file 1 — Supplementary Information [file 41598_2019_50587_MOESM1_ESM.pdf]

ARA: accurate, reliable and active histopathological image classification framework with Bayesian deep learning
Supplementary Information

Alicja Rączkowska[1], Marcin Możejko[1], Joanna Zambonelli[2], and Ewa Szczurek[1,*]

[1]Faculty of Mathematics, Informatics and Mechanics, University of Warsaw, Warsaw, Poland
[2]Department of Pathology, Medical University of Warsaw, Warsaw, Poland
[*]szczurek@mimuw.edu.pl

Network architecture

The ARA-CNN network accepts RGB images of size $(128,128,3)$ as its input (Fig. S1A), where the values represent respectively: vertical resolution, horizontal resolution and the number of colour channels. The images from the training dataset were downsized to these dimensions. Input values are propagated to the first part of the network called stem (Fig. S1B). The stem contains a convolutional layer consisting of 64 filters, with filter size of $(7,7)$ and stride size of $(4,4)$. This is directly followed by max pooling with window size of $(2,2)$, and identically sized strides. The output from this part is of size $(16,16,64)$, where the values are: reduced width, reduced height and the number of filters. These operations decrease the spatial dimensions by a factor of 8, which in turn significantly reduces memory usage and can be considered an adaptation of network topology to a relatively simple texture structure of the input[1].

The stem is followed by the first block (Fig. S1C). The main aim of this part is to learn and extract initial discriminative low-level image features. It consists of 4 residual sections, where the input to each block is transformed by a convolutional layer with 64 filters - each sized $(3,3)$ and with stride of size $(1,1)$. The result of this convolution is added back to the input, which creates a residual connection. The final section of this block is followed by an average pooling with window size of $(2,2)$. This makes the output of this part of the network to be shaped $(8,8,64)$. The next part of the model is the second block (Fig. S1D), which learns and extracts the final discriminative features - this time they are more high-level and abstract. Its structure is the same as that of the first block. After the final average pooling with window size $(2,2)$, the output from this part is of size $(4,4,64)$.

The model has two outputs in total: the auxiliary output (Fig. S1E) and the main output (Fig. S1F). The main purposes of the former are to provide a better training signal to the stem and the first block (by making the features more discriminative) and to deal with the vanishing gradient problem[2] during training. The output from the first block is transformed by global average pooling. Next, it is transformed by a fully-connected layer with 32 filters and dropout with rate of 0.5 (explained in *Dropout*, see main text). Finally, it is fed to the fully-connected output layer with a softmax activation function. The final output is used for making the actual predictions. After the second block, the data is transformed by exactly the same set of transformations as in the auxiliary output - global average pooling, then a fully-connected layer with dropout, followed by a final output layer.

If not stated otherwise, each convolutional filter has dilation and stride set to 1. Additionally, each layer in the network (except the outputs) connects to a Batch Normalization layer. When deep learning models are trained, the distribution of inputs for each layer changes as a result of modified parameters in preceding layers, which slows down the whole process. Batch Normalisation combats this by normalising layer inputs for each training mini-batch. This enables the use of higher learning rates and significantly speeds up the training. Batch Normalisation also acts as a regulariser and reduces overfitting. The activation function used throughout the model is Leaky ReLU[3]:

$$f(x) = \begin{cases} x & \text{for } x > 0 \\ \alpha x & \text{otherwise,} \end{cases} \quad (1)$$

where the parameter α is set to 0.1 and x is a weighted sum of inputs to a network unit.

Tissue slide segmentation

In histological image analysis, the labelling of image patches is only the first step in the process of segmentation. To get a full overview of a tissue slide, it is necessary to see how image patches of different classes are placed in relation to each other and

to measure their relative abundance. In particular, it is interesting to determine the neighbourhood of tumour cells. For example, the tumour being infiltrated by immune cells may be a marker of good prognosis. *Kather et al.*[4] showed a simple segmentation approach using standard classification methods. We present a recreation of their procedure using the ARA-CNN model (see *Image segmentation* in Methods).

An example segmentation of five full tissue slides from the *Kather et al.*[4] dataset is presented in Fig. S2. The segmentation can obviously be improved - the approach with stitching image patches is after all quite rudimentary. However, it can be good enough to see the aforementioned spatial relationships. As a basic spatial statistic, for each slide we generated a summary of tissue class distribution (Fig. S2). Histograms such as these can be used as a filter to find images for further consideration (e.g. those with high tumour concentration) in an automated diagnosis system.

References

1. Goodfellow, I., Bengio, Y. & Courville, A. *Deep Learning* (MIT Press, 2016).
2. Hochreiter, S., Bengio, Y., Frasconi, P. & Schmidhuber, J. Gradient flow in recurrent nets: the difficulty of learning long-term dependencies. *A Field Guid. to Dyn. Recurr. Neural Networks* (2001).
3. Xu, B., Wang, N., Chen, T. & Li, M. Empirical evaluation of rectified activations in convolutional network. *CoRR* **abs/1505.00853** (2015).
4. Kather, J. N. *et al.* Multi-class texture analysis in colorectal cancer histology. *Sci. Reports* (2016).

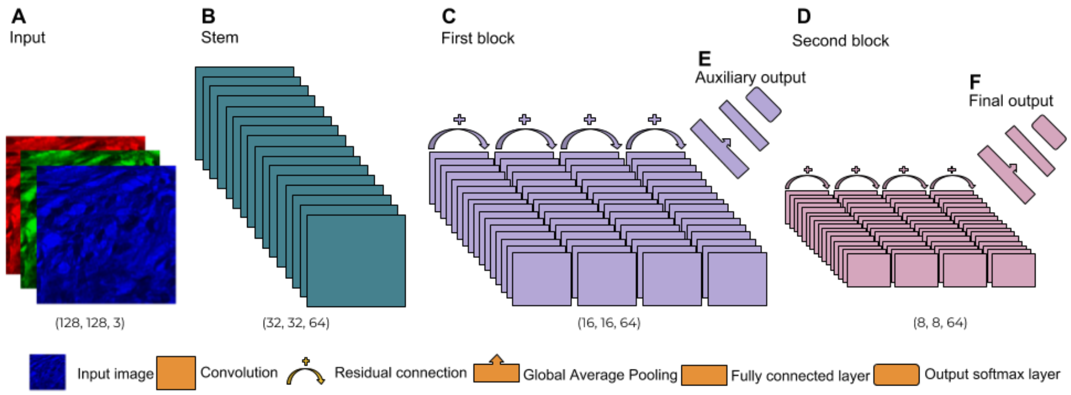

Figure S1. Structure of the ARA-CNN model. The network takes as input RGB images with dimensions of 128x128 pixels. They are passed to the stem, which contains a convolutional layer responsible for reducing the spatial dimensions of the input. This is followed by the first block and the second block, responsible for learning low-level and high-level image features respectively. Both of these blocks consist of four residual sections, with each of these sections containing a convolutional layer and a residual connection. The model has two outputs overall - an auxiliary output from the first block and a final output from the second block. Both of them use the softmax activation function.

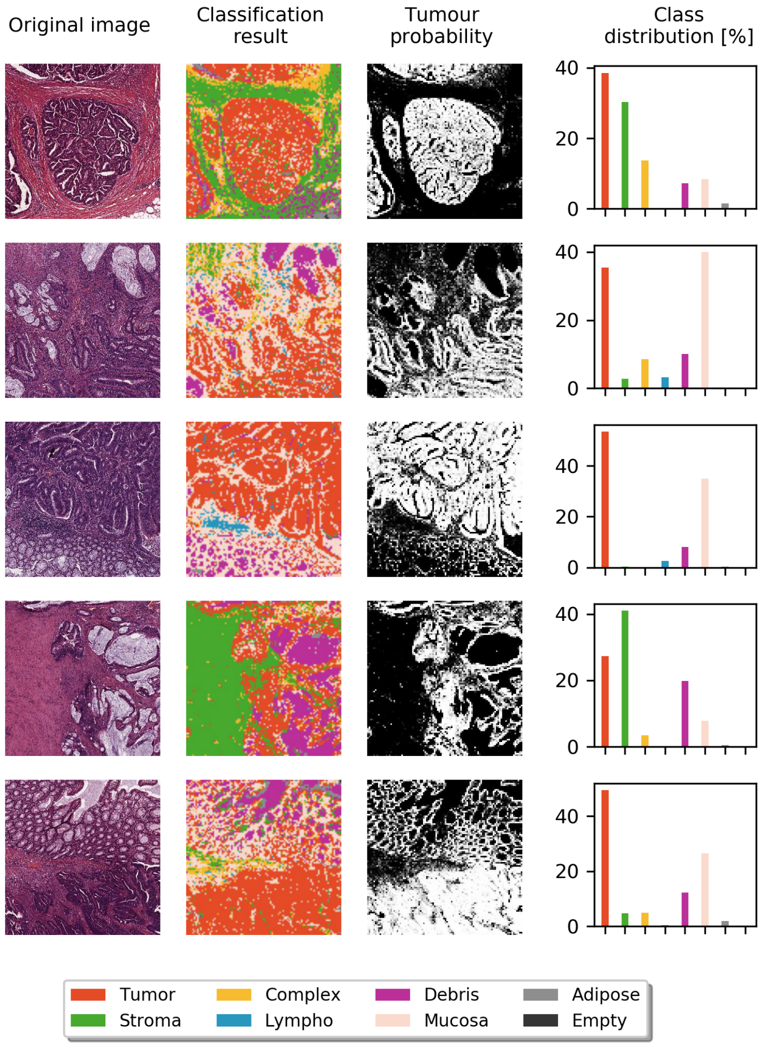

Figure S2. Segmentation. Segmentation of five large tissue slides from the colorectal cancer dataset. The leftmost column presents the original WSIs, the second one shows the segmentation done with our classification algorithm, while the third one is a visualisation of the Tumour class probability (the lighter the segment, the more probable that there is a tumour there). The last column contains a class distribution histogram - each bar represents the percentage of a given class in the segmented image.
